# The Role of BPIFB4 in Immune System and Cardiovascular Disease: The Lesson from Centenarians

**DOI:** 10.37825/2239-9754.1029

**Published:** 2021-12-23

**Authors:** Francesco Montella, Valentina Lopardo, Monica Cattaneo, Albino Carrizzo, Carmine Vecchione, Elena Ciaglia, Annibale Alessandro Puca

**Affiliations:** a Department of Medicine, Surgery and Dentistry “Scuola Medica Salernitana”, University of Salerno, Via Salvatore Allende, 84081, Baronissi, Salerno, Italy; b Cardiovascular Research Unit, IRCCS MultiMedica, 20138, Milan, Italy; c Department of Vascular Physiopathology, IRCCS Neuromed, Pozzilli, 86077, Isernia, Italy

## Abstract

Recent discoveries have shed light on the participation of the immune system in the physio pathology of the cardiovascular system underpinning the importance of keeping the balance of the first to preserve the latter. Aging, along with other risk factors, can challenge such balance triggering the onset of cardiovascular diseases.

Among several mediators ensuring the proper cross-talk between the two systems, bactericidal/permeability-increasing fold-containing family B member 4 (BPIFB4) has been shown to have a pivotal role, also by sustaining important signals such as eNOS and PKC-alpha.

In addition, the Longevity-associated variant (LAV), which is an haplotype allele in BPIFB4 characterized by 4 missense polymorphisms, enriched in homozygosity in Long Living Individuals (LLIs), has been shown to be efficient, if administered systemically through gene therapy, in improving many aspects of cardiovascular diseases (CVDs). This occurs mainly through a fine immune system remodeling across: 1) a M2 macrophage polarizing effect, 2) a favorable redistribution of the circulating monocyte cell subsets and 3) the reduction of T-cell activation. Furthermore, LAV-BPIFB4 treatment induced a desirable recovery of the inflammatory balance by mitigating the pro-inflammatory factor levels and enhancing the anti-inflammatory boost through a mechanism that is partially dependent on SDF-1/CXCR4 axis.

Importantly, the remarkable effects of LAV-BPIFB4 treatment, which translates in increased BPIFB4 circulating levels, mirror what occurs in long-living individuals (LLIs) in whom the high circulating levels of BPIFB4 are protective from age-related and CVDs and emphasize the reason why LLIs are considered a model of successful aging. Here, we review the mechanisms by which LAV-BPIFB4 exerts its immunomodulatory activity in improving the cardiovascular-immune system dialogue that might strengthen its role as a key mediator in CVDs.

## 1. Introduction

**Fig. 1:**
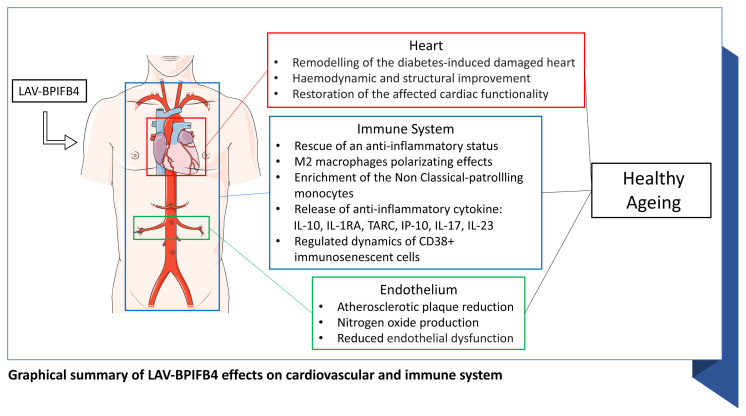
LAV-BPIFB4 effects on cardiovascular and immune system. LAV-BPIFB4 can influence the activity of immune cells in atherosclerosis, diabetic cardiomyopathy, and cardiovascular complications mainly by remodelling heart, enhancing anti-inflammatory circuits and prevent endothelium dysfunction.

CVDs represent the leading cause of death worldwide. According to WHO, 17.9 million people died from CVDs in 2019 (about 32% of global deaths) [1]. In order to ameliorate the management of CVDs, a more integrated vision of cardiac biology is critical to better determine the mechanisms leading to CVDs onset and progression. Not surprising the cardiovascular system is made up of a wide range of cells that interoperate generating a complex network capable in maintaining the entire organism functionality and integrity. In heart, ventricular and atrial cardiomyocytes represent the most abundant cell population followed by endothelial cells, fibroblasts, pericytes, smooth muscle cells, adipocytes, mesothelial cells, neuronal cells and - resident or from circulating blood - immune cells of both myeloid (monocytes, macrophages, neutrophils and dendritic cells) and lymphoid origin (T and B cells) [2]. Even if immune cells represent the minor population of the heart (between 5% and 10%), their involvement in both physiological and pathological conditions in heart and their dialogue with myocytes and non-myocytes cells is definitely an interesting field of study. Endothelial cells, smooth muscle cells and pericytes are the major cellular component of vessels, arteries, and capillaries and interact through a bidirectional interchange of signals and factors with circulating immune players [3,4]. These intricate cross-talks maintain the myocardium and vessels homeostasis and, when unbalanced, lead to several CVDs. This unbalance occurs during aging thus triggering the onset of CVDs. The remodeling of the morphological structure of heart and vasculature, the persistent exposure to risk factors together with the progressive age-associated decline in heart functionality and integrity, result in several cardiovascular diseases, including atherosclerosis, hypertension, diabetic cardiomyopathy, myocardial infarction, stroke and others [5]. Compared to elderly people, long-living individuals (LLIs) represent a model of successful healthy aging. LLIs are less prone to be affected by CVDs thanks to their ability in preserving both immune response and inflammatory balance, which are usually lost and/or deteriorated in the elderly. Here we will discuss the role of the LAV haplotype in the BPIFB4 gene found enriched in homozygosity in LLIs and under positive and balancing selection, in restoring the proper inflammatory balance and successfully modulating the immune system in physio-pathological conditions directly (or indirectly) related to CVDs. (see Fig. 1).

## 2. BPIFB4 and its longevity-associated variant: from the expression in long-living individuals (LLIs) to the protective mechanisms in disease

### 2.1. BPIFB4-related immunomodulatory effects in LLIs

Despite the increase of life expectancy in industrialized populations that occurred in the last century, a sedentary lifestyle, wrong nutritional habits and exposure to pollution led to an increased incidence of CVD-related morbidity and mortality. The case of Long-Living Individuals (LLIs) is the unique model of delaying or avoiding diseases of aging, mainly CVDs, extending life-span of 40 years in comparison to their original cohort life expectancy. While it is true that diet and exercise are impacting on the ability to reach eighties in good health, there is no external intervention that correlates with succeeding in reaching extreme ages, such as the supercentenarian ones (110 years old). Thus, despite many evidences are pointing to a balanced diet, such as Mediterranean Diet, which is characterized by a low glycemic index (GI), a low animal protein intake, phytochemical compounds (found in vegetables, fruit, red wine, olive oil) as protective towards cancer and cardiovascular diseases [6], genetic factors may represent a main cause for the increased average survival rate of LLIs. Indeed, LLIs are gifted by a genetic signature that allow them to survive to extreme ages avoiding diseases of aging [7]. This reflects in exceptional longevity occurring in families, with a reduction of CVDs and overall mortality among centenarian’s offspring and siblings [8–10]. In the recent years GWASs (Genome Wide Association Studies) have been run on LLIs and controls to identify genetic variants that confer either advantage or a disadvantage to reach extreme ages. LAV-BPIFB4 is a four-missense single-nucleotide polymorphism haplotype of the Bactericidal/permeability-increasing fold-containing family B member 4 (BPIFB4) gene that has been found to be enriched as homozygous genotype in LLIs, thus protective for human health, in three independent populations from Italy, Germany and USA [11] with an allele frequency of 29.5% with respect to the 66% of the Wild Type (WT) haplotype. LAV homozygous genotype reaches 14% in centenarians compared to 10% in controls [11–13]. The homozygous genotype showed a clear correlation with high eNOS phosphorylation in serine 1177 that correlates with high activity and Nitric Oxide (NO) production in PBMCs of healthy subjects. Furthermore, an inverse correlation of the homozygous LAV-BPIFB4 was found with frailty in elderly subjects and accordingly, in old mice LAV-BPIFB4 gene transfer delayed frailty progression [13]. BPIFB4 is a secreted protein found in respiratory secretion, upper airways and proximal trachea [14] with an appreciable expression also in cardiomyocytes and endothelial cells [15], PBMCs [16], cardiac pericytes (personal communication) and striatal-derived cell lines [17]. BPIFB4 is particularly abundant in serum of long-living individuals presumably allowing them to maintain a proper balance between anti-inflammatory and proinflammatory mechanisms without encountering the detrimental remodeling of immune system usually found in the elderly [18].

The genetic and epigenetic findings described above about BPIFB4 and LLIs, gave an impulse to further scan LLIs for the identification of new molecules to be candidates for their delaying of CVD and other age correlated diseases. A cytokines analysis conducted in a first cohort of N = 20 Long living individuals (LLIs) from Cilento, a rural area of the South Italy, demonstrated an increase of IL-10 and IL-1RA in the plasma of LLIs compared to healthy volunteers [19]. From a functional point of view, we also recently described a peculiar anti-inflammatory ability of LLI’s plasma. When healthy monocytes-derived macrophages were differentiated in presence of plasma from LLIs, they acquired a CD206 + CD163+ macrophage phenotype, highlighting their M2 anti-inflammatory nature. Furthermore, BPIFB4 protein, found enriched in LLIs’ plasma, was responsible for the M2 polarizing activity of LLIs’ plasma. Indeed, the BPIFB4-neutralizing antibody induced a significant decrease in macrophage M2 recovery upon stimulation with LLIs’ plasma [20]. Next, a Multiplex bead-based immunoassay on LLIs’ plasma revealed that other factors characterized LLIs compared to adult individuals. Among these, thymus and activation regulated chemokine (TARC)/CCL17 and small-inducible cytokine B10 (IP-10)/CXCL10) cytokines were the only additional factors enriched in centenarians, thus indicating them as additional key mediators of successful ageing [21]. The two cytokines are mainly released from myeloid cells: IP-10 is an effector cytokine enhancing host performance against infectious disease [22]; TARC is implicated in the T cell recruitment and produced from M2 macrophages as effector molecule that inhibit M1 inflammatory macrophages generation [23]. However only TARC seems to be responsible for M2 polarizing action of LLIs plasma as TARC-, but not IP-10-blocking antibodies were able to rescue the M2 enrichment in monocyte-derived macrophage compartment [21].

Taking all the above results in consideration, we reasoned that the peculiar cytokine profile may be responsible for the skewed monocytes asset found in LLIs. Indeed, characterization of circulating monocytes from 52 LLIs (median-age 97) and 52 healthy volunteers (median-age 55) showed that the intermediate CD14++CD16+ monocytes counts were lower in LLIs compared to control adults. Conversely, non-classical CD14 + CD16++ monocyte counts, which are M2 macrophage precursors with an immunomodulatory function, were found significantly associated with the LLIs’ state [21].

The enriched subset of non-classical monocytes is known to actively patrol the vasculature and remove damaged cells in several disease conditions, thereby aiding tissue healing and the resolution of inflammation [24]. Further, non-classical patrolling monocytes might serve as precursors for protective M2-macrophages during soft tissue injury [25].

### 2.2. Molecular events regulating BPIFB4 action

Mechanistic investigations elucidated the signal transduction pathways by which BPIFB4 protein, and its LAV-isoform induces pleiotropic effects on immune system or in presence of cardiovascular disease to offer protection from age-related impairment. Among these, we previously reported that protein kinase R-like endoplasmic reticulum kinase (PERK), a kinase involved in the unfolded protein response, exerts the phosphorylation at Serine 75 of BPIFB4 (that seems to have a cytoplasmatic localization), and its LAV-variant in endoplasmic reticulum stress response and cellular homeostasis. This event also allows its interaction with the 14-3-3 protein and the Heat Shock Protein 90 (HSP-90) resulting in the BPIFB4-14-3-3-HSP90 complex that induces eNOS phosphorylation and NO production in endothelial cells [26]. To be noted, the formation of the complex that is paired with the BPIFB4 serine 75 phosphorylation, is a peculiarity of LAV-BPIFB4. Consistently, ectopic expression of LAV-BPIFB4 in HUVEC endothelial cell lines resulted in the stimulation of NO production due to the activation of the Protein kinase C alpha (PKCα) signaling. Mechanistically, LAV-BPIFB4 augmented Ca^2^^+^ mobilization that in turn increased the translocation of PKCa to the plasma membrane. Importantly, in the absence of external Ca^2^^+^, the ability of LAV-BPIFB4 to enhance both PKCa and eNOS phosphorylation was abolished [27]. More recently, gene therapy in ApoE knockout mice with AAV-LAV-BPIFB4 has been described to reduce the formation of vascular plaques, and this effect was lost by the co-treatment with AMD3100, an inhibitor of CXCR4 receptor (a seven transmembrane G-protein-coupled, alpha-chemokine receptor specific for SDF-1α), which suggests a possible involvement in the LAV-BPIFB4 regulation of calcium via CXCR4-SDF1 pathway [28]. The effects of LAV-BPIFB4 on this axis was valid also in the brain. Indeed, in a mouse model of Huntington’s disease, the LAV-BPIFB4 plays a critical role in a CXCR4-dependent action, as shown both in vitro and in vivo, inducing SDF-1 expression from striatal cells and polarizing human microglia to an antiinflammatory status in culture. In this context the ability of LAV-BPIFB4 to finely shape the M1–M2 balance of human microglia in part explains its therapeutic potential [17].

## 3. The auspicable atheroprotective role of LAV-BPIFB4 in human patients and in a mouse model of the disease

### 3.1. Atherosclerosis physiopathology

Atherosclerosis is a chronic inflammatory disease and it is considered maybe the first cause of coronary artery disease and stroke [29]. Generally, atherosclerosis was considered a cholesterol storage disease caused by the accumulation of lipoproteins including LDL in the intimal tissue of arteries resulting in the continuous growth of fatty infiltrates rich in inflammatory leukocytes that macroscopically appear as plaques [30]. Plaque ruptures at the surface trigger thrombosis with a huge involvement of platelets [31]. The role of the innate immune system especially monocytes and macrophages, together with neutrophils and lymphocytes are also key for the onset and development of atherosclerosis [32,33]. Resident macrophages in atherosclerotic lesions are continuously exposed to accumulating lipids and their oxidized derivatives. Cholesterol clusters and oxidized LDL can induce monocytes and macrophage to polarize in inflammatory cells. Thus, in response to this environment, macrophages adapt their phenotype to a M1 activation state [34]. M1 macrophages are associated with symptomatic and unstable plaques, whereas M2 macrophages are particularly abundant in stable zones of the plaque and asymptomatic lesions. This suggests that M2 macrophages are localized within human plaques and their intrinsic anti-inflammatory properties, are primarily associated with plaque stability [35,36].

### 3.2. LAV-BPIFB4 role in atherosclerotic process

The balance between the thickness of fibrotic cup that surrounds the plaque and the inflammatory status determines the stability of the entire system. An unbalanced environment leads to a break of the plaque with the high risk of circulating debris and thrombus with consequential ictus, myocardial infarction or stroke [37]. In this scenario, LAV-BPIFB4 was found to reduce atherosclerotic progression leading the immune system to an anti-inflammatory polarization. In an ApoE knockout mice fed a high-lipid diet, a well-known atherosclerosisprone murine model, it has been demonstrated the efficacy of LAV-BPIFB4 in contrasting endothelial dysfunction, plaque progression, inflammatory cytokine release, macrophage shift, and T cell activation. First we observed that LAV-BPIFB4 gene transfer causes a CXCR4-dependent redistribution of the two major monocyte subsets with reduced Ly6Clow subsets and increased circulating Ly6Chigh cells, common precursor of M2 atheroprotective macrophages [28]. As a result, LAV-BPIFB4 gene therapy reduced the proliferation of Ki-67+ T cell in murine blood. Looking at CD3+ subset, LAV-BPIFB4 gene decreased the abundance of proliferating cytotoxic CD8+ T. These effects were inhibited by the CXCR4 inhibitor AMD3100. Following a translational approach, we proved that the healthy phenotype of LLIs can be transferred to both murine models and cultured human tissues not only by the delivery of LAV-BPIFB4 gene but also by a recombinant protein (rhLAV-BPIFB4) treatment. Indeed, the administration of recombinant protein lead the atherosclerotic patients macrophages to acquire a M2 phenotype, as shown by the up-regulation of the canonical M2 marker CD163. Furthermore, the LAV-BPIFB4 recombinant protein finely modulated the cytokine secretion from atherosclerotic vessel after 72 h of culture, increasing the atheroprotective IL-33 and down-regulating pro-inflammatory IL-1β and TNF-α. In this view, LAV-BPIFB4 appears as a promising immunoregulatory agent in atherosclerosis. Indeed, LAV-BPIFB4 gene therapy succeeded in the two primary endpoints: improving endothelial dysfunction and reducing adverse vascular effects in ApoE knockout mice [28]. More interestingly, from a clinical point of view, in patients with no subclinical carotid atherosclerosis, the protein’s concentration was significantly higher if compared to patients with carotid stenosis. Furthermore, genotype stratification analysis confirmed that LAV carriers have more frequently an intima media thickness (IMT) < 2 mm and had a higher level of protective BPIFB4 compared to WT carriers [28]. Overall these preclinical data proposed LAV-BPIFB4 as relevant immunomodulatory/immunotherapeutic tool in Atherosclerotic progression.

## 4. The role of LAV-BPIFB4 in rescuing heart functionality in diabetic cardiomyopathy

Diabetic cardiomyopathy is a clinical condition of myocardial dysfunction that occur in diabetic patients without cardiac risk factors (ie, coronary artery disease, hypertension, valvular complications), which in turn becomes a risk factor for heart failure [38,39].

From a metabolic point of view, hyperglycaemia, hyperlipidaemia and lipotoxicity caused by a lack of insulin or insulin resistance trigger mithocondrial dysfunction due to increased intracellular fatty acid, enhanced ROS and RNS production. These events in turn lead to cardiac alterations: 1) cardiomyocytes death and hypertrophy, 2) cardiac fibrosis associated with impaired ECM degradation by MMPs, release of TNF, TGF-β, angiotensin II 3)microvascular dysfunction mediated by endothelial damage (reduced NO signalling) [40]. Pro-inflammatory pathways work synergically with the pathological mechanisms of diabetic cardiomyopathy leading to a systemic and cardiac inflammatory cell activation, as shown by the high detrimental levels of IL-1β, NF-kb, TNF-α, AKT1 and lypoxigenase. At this regard, we have been recently investigated the immunomodulatory role of LAV-BPIFB4 in diabetic cardiomyopathy [15] through the SDF-1/CXCR4 axis, known to be involved in the development of cardiac dysfunction underpinning diabetic cardiomyopathy [41,42] and reported as the mechanisms through which LAV-BPIFB4 induces M2 macrophage polarization in atherosclerosis [28]. Both in human cardiac cells and in validated db/db mice model, the overexpression of LAV-BPIFB4 had a desirable effect in restoring the affected cardiac functionality compromised by the diabetes-induced damage. Indeed, LAV-BPIFB4-treated mice showed an improved systolic function and capillaries density coupled with the increased expression of MyHC-α, involved in the contractile property of myocytes and endothelial function, and reduction in heart lipid content. The in vivo co-treatment with LAV-BPIFB4 and an antagonist of CXCR4 (ie, AMD-070) prevented the resolutive effect of LAV-BPIFB4 confirming that this peculiar isoform of BPIFB4 operates through SDF-1/CXCR4 axis. LAV-BPIFB4 in vitro treatment and in vivo gene therapy were also able to induce the expression of SDF-1 respectively in human CD14 + CD16+ intermediate monocytes from type 2 diabetic patients and in peripheral blood and heart of diabetic mice. In concert with the beneficial effect of SDF-1 on heart and the ability of BPIFB4 in promoting the SDF-1 expression on monocytes and in db/db mice heart, LAV-BPIFB4 might be considered as an immunoregulatory driver in CVDs (i.e. diabetic cardiomyopathy) considering its influence on SDF-1 expression that in turn could exhibit its protective role.

## 5. BPIFB4 as a prognostic factor in COVID-19

Cardiovascular diseases constitute one of the most common comorbitities (with a prevalence of 8% from independent studies) [43,44] in patients infected with Severe acute respiratory syndrome coronavirus 2 (SARS-CoV-2), the Coronaviridae family virus causing COVID-19 pandemic. CVDs are doubly implicated in COVID-19 both due to the high risk of COVID-19 in pre-existing CVDs patients and on account of the cardiovascular implications of the infection among patients with COVID-19 [45]. Briefly, the viral spike protein binds the ACE2 receptor expressed on host cells and, following the entry and replication, it triggers an uncontrolled immune response to which the immune system responds with an inadequate virus clearance and an inflammatory imbalance that conversely leads to the sadly known “cytokine storm”. The Angiotensin-converting enzyme 2 receptor is not only expressed on nasal epithelial cells, lungs, and bronchial branches (making ACE2 receptor the key factor for SARS-CoV-2 infection) but also on cardiomyocytes, cardiofibroblasts, pericytes, coronary endothelial cells and cardiac muscle cells [46]. The almost ubiquitous presence of ACE2 receptor on different cardiac cell subtypes might explain the potential involvement of the cardiovascular system in SARS-CoV-2 infection and the related complications including myocarditis, heart failure, cardiomyopathy, myocardial infarction, and thromboembolic events [47]. In addition, the dysregulation of cardiac immune cells unabled to counteract the infection and the cytokine storm established the need to investigate successful immunotherapies for COVID-19 and cardiovascular complications. Among the others (recently reviewd by Xie et al., 2021, [48]), BPIFB4 seems to be a valid prognostic marker for COVID-19 being its low circulating levels in SARS-CoV-2-positive individuals (with respect to LLIs and controls groups) that is further reduced in high-grade patients [49]. In vitro experiments on PBMCs have showed a biphasic immune response mediated by recombinant LAV-BPIFB4 (rLAV-BPIFB4) that in the first phase of the infection induced a hyperactivation of CD4+ and CD8+ lymphocytes that decreases in the ending phase of the treatment demonstrating that this longevity-associated variant has a considerable immune shaping activity. In advanced heart failure, the chronic activation of lymphocytes is responsible of cardiovascular insult [50]. Hence the two-phase immune response induced by rhLAV-BPIFB4 in COVID-19 context might be crucial in remodeling the perpetuated lymphocytes activation involving in heart failure. Coupled with this dual immunomodulatory activity on lymphoid compartment, rhLAV-BPIFB4 was able to modulate the PBMCs microenvironment inducing the reduction of MCP-1 levels accompanied by higher amount of IL-1β and IL-18 required for the immune response against the virus. MCP-1 is well-known for its role in regulating monocytes chemotaxis into the situ of inflammation and T-lymphocytes differentiation both in health and disease [51]. In light of that, MCP-1 (and its binding to cells expressing CCR2) has a pivotal role in atherosclerosis and myocardial infarction [52,53]. Mediators of CVDs can induce a significant release of MCP-1 by endothelial cells and smooth muscle cells of vascular wall promoting monocyte diapedesis into the endothelial space and the accumulation of lipid foam cells in the arterial wall. Since the crucial role of endothelial cells in COVID-19 and the known role of LAV-BPIFB4 in modulating the endothelial function [11,54], BPIFB4 might exert its protective effect in endothelium dysfunction occurring during inflammatory conditions also opposing high levels of MCP-1.

Compared to inflammaging occurring in elderly people, LLIs maintain a fine balance between anti-inflammatory and pro-inflammatory mechanisms and the Longevity-associated variant of BPIFB4 has proven to ensure the adequate inflammatory response in the above-investigated experimental setting of COVID-19. Its potential role as positive prognostic marker in COVID-19 is also confirmed by a better response to SARS-CoV-2 infection registered in centenarians of Italian region [55].

## 6. The involvement of BPIFB4 in immune response to prolonged infections

Bacteria, fungi, viruses, and parasites can infect the organism in diverse sites such as lung, bloodstream, renal and genitourinary tracts. Stimulation of pattern recognition receptors (PRRs; ie, TLRs, NLRs, CLRs) by molecular patterns from microbial components named PAMPs (ie, LPS) triggers the innate immune response through the activation of signaling pathways (ie, NF-kb, JNK, ERK, inflammasome activation) that elicit the initial hyper-inflammatory response (made up of an intricate interplay of pro-inflammatory and anti-inflammatory processes) followed by a considerable immunesuppresion phase [56,57] leading to a generalized dysregulation of the immune response. Neutrophils are systematically activated showing both an impaired migration in infection sites and increased release of immature neutrophils in blood stream after augmented granulopoiesis process [58]. Monocytes from septic patients show decreased HLA-DR and CD86 levels coupled with decreased expression of CD163 and increased expression of CD206 receptors [59,60]. Taken together these data suggest a dysregulated antigen presentation activity accompanied by higher propensity to acquire an anti-inflammatory phenotype demonstrating a partial anergy in monocytes during uncontrolled host response to infections. Monocytes migrate rapidly during acute inflammation and can differentiate into macrophages or dendritic cells [61]. Dendritic cells undergo a caspase-3-mediated apoptosis and an excessive activation due to systemic circulation of PAMPs resulting in a limited ability of DCs in priming T cell response [62]. If macrophages acquire a M1-like phenotype aimed to eliminate the pathogens in the early phase of infections, they quickly switch in a state of damaging tolerance towards the infectious agent (eg, LPS) leading to T cell apoptosis and the suppression of Th1 response [63]. As above mentioned, BPIFB4 is particularly abundant on olfactory epithelium, in respiratory secretions, upper airways and proximal trachea [64]. Seen its expression in several hypothetical site of infection, its role as both defensine and immune-driver has been explored. LPS-infected human CD14+ monocytes and induced-dendritic cells have demonstrated to be less prone to release TNF-α and IL-1β when pretreated with LAV-BPIFB4 [20] re-confirming the ability of the protein variant in balancing the inflammatory microenvironment. At the same time, monocyte-derived DCs exposed to LAV-BPIFB4 secreted more anti-inflammatory factors as IL-10 and TGF-β in response to LPS and higher expression of CD11b marker tolerogenic. These data suggest LAV-BPIFB4 is able to blunt the proinflammatory response to LPS in monocytes and dendritic cells, the major innate immune cells involved in struggling infections.

Due to the diminished immune system efficiency (termed immune senescence), the persistence of a chronic low-grade pro-inflammatory state and comorbidities occurrence [65], elderly people display a decreased ability in clearing infections and in managing their clinical complications. In agreement with the above-mentioned in vitro observations, long-living individuals show a peculiar cytokine profile enriched with IL-10 and IL-1RA compared to healthy adults. This finding could lay the basis to speculate that the strong anti-inflammatory environment of LLIs is able to counterbalance the low-grade inflammatory background that makes elderly people less able to fight against infections.

## 7. A putative senotherapeutic action for LAV-BPIFB4

It is widely known that the incidence of CVDs increases with aging [66]. This higher prevalence of CVDs in elderly population has been linked to several events among which a paradox has been contemplated as the major hallmark of ageing. Indeed, the gradual decline in immune cells function and abundance (hence insufficiency and dysregulation, termed immunosenescence, [67]) triggers a persistent chronic low-grade inflammation (hence over-reaction, termed inflammaging, [68]). Anatomically, the thimic involution occurring during aging produces ineffective central tolerance and reduced thymopoiesis leading to increased autoimmune responses and decreased efficiency in fighting infections. In addition, senescent cells release SASP factors (i.e., cytokines, chemokines, matrix metalloproteases, growth factors) influencing neighbouring cells and contributing to inflammaging. Even if acute cellular senescence has a beneficial role in heart regeneration and development [69], emerging data have proven that during aging the long-term accumulation of senescent cardiovascular and immune cells lays the basis for pathogenesis of several CVDs, such as hypertension [70], atherosclerosis [71,72] and heart failure [73]. In this regard, the role of LAV-BPIFB4 in delaying or halting immunosenescence has been investigated.

As the intricate cross-talk between cardiac cells and immune cells preserves cardiovascular homeostasis, immune senescent cells could contribute to enhance CVDs along with senescent myocytes, mainly in elderly [74,75]. To this aim, our data showed AAV-LAV-BPIFB4 gene transfer may counteract immunosenescence and vasculature aging in old mice [76]. Firstly, we found a significant decrease in SA-β-Gal activity in CD11b + myeloid cells isolated from 26 months C57BL/6J mice receiving the AAV-LAV-BPIFB4 compared to old-GFP mice. Furthermore, plasma of AAV-LAV-BPIFB4 mice showed a better SASP factor profile made up of lower levels of IL-1β and higher IL-10 levels. As immune senescent cells can regulate the myocardial microenvironment also by directly modulating myocytes senescence [77], these data suggest LAV-BPIFB4 gene transfer may be a valid tool in educating the senescence phenotype associated to worse CVDs outcomes. Since senescent cells release SASP factors in a non-cell autonomous manner eliciting a response in surrounding cells and tissues [78], aortas from old-mice showed a higher percentage of SA-b-Gal positive area that was significantly reduced in response to AAV-LAV-BPIFB4 in vivo treatment. Collectively, these data demonstrated LAV-BPIFB4 could shape the inflammatory cytokine plethora induced by senescence that in turn leads to tissue damage and ageing.

Recently, senescence and inflammation in ageing have been associated to NAD + levels decline attributable to higher expression of NAD + consuming enzymes, including CD38, in immune cells compartment [79]. Our data showed that AAV-LAV-BPIFB4 treatment induces a recovery in NAD + levels comparing to young and untreated mice. This data reached a translational value considering that LLIs showed a statistically significant increase of plasmatic NAD + levels with respect to old healthy control demonstrating LAVBPIFB4 can positively interfere with the NAD + - related ageing process in vivo. Moreover, LLIs genotype analysis proved that LAV-carriers are characterized by higher levels of NAD+ in comparison to WT-carriers. In this light, this could be added to all those reason why LLIs and LAV-BPIFB4 carriers are less inclined to develop age-related diseases. Moreover, the AAV-LAV-BPIFB4 treatment seemed to provide a very efficient catabolic activity in the spleen of aged mice. Indeed, AAV-LAV-BPIFB4 leads to the enrichment of senescent cells in spleen coupled with the increase of active NK1.1 + CD69+ cells among total splenocytes suggesting a role of LAV-BPIFB4 in clearing senescent cells through NK-induced cytotoxicity. Remarkably, AAV-LAV-BPIFB4 gene transfer also increased the M2 proresolving macrophages compartment in the spleen, reduced the amount of both spleen-resident CD38+ macrophages and CD38+ pro-inflammatory monocytes and mitigated the splenic inflammatory microenvironment (generating a milieu made up of lower levels of IL-1β, IL-6, IFN-γ and higher levels of IL-10).

To improve matters, the positively arranged milieu of AAV-LAV-BPIFB4 mice was capable to in vitro shaping the immune asset of a monocyte/macrophage cell line by inducing the acquisition of M2-like phenotype and the internalization (and not the surface upregulation) of CD38 marker demonstrating AAV-LAV-BPIFB4 may be able in counter-acting the non-autonomous effect by which senescence is gained by non-senescent cells. The role of LAV-BPIFB4 in enhancing the catabolic activity of the main hemocateretic organ and in restoring the activation of clearance mechanisms lost during ageing could be very beneficial in the reduction of cardiovascular senescence cell burden profoundly connected to a variety of CVDs [80]. Moreover, the anti-inflammatory microenvironment opposing the SASP, the M2 polarizing effect (that in heart have pro-fibrotic and pro-angiogenic properties, [81]), the modulation of CD38 expression and NAD + activity (well known to be involved in several CVDs, as hypertension, arrhythmia, coronary heart disease, [82]) elect LAV-BPIFB4 as valid tool in delaying heart senescence as well done in immune system.

As the excessive senescent cells accumulation represents one of the pillars of ageing and chronic diseases [83,84], AAV-LAV-BPIFB4 treatment showed both catabolic and senotherapeutic effects by succeeding in proper eliminating and shaping senescent cells. Seen the role of LAV-BPIFB4 in rejuvenating both the immune and cardiac senescent compartment and considering the involvement of CD38-dependent NADase activity in cardiac myocytes and endothelial cells [85], a potential role of LAV-BPIFB4 in reducing the CD38 activity in myocyte cells is plausible. Moreover, the peculiar attitude of LLIs in stemming the senescence process is another lesson to be considered for better exploring ageing and age-related disease. In this perspective, LAV-BPIFB4 could be a useful senotherapeutic agent in heart and immune system ensuring the homeostasis lost with ageing.

## 8. Conclusions

A plethora of research papers have been clearly documented the activation of immune pathways and the involvement of both cellular and soluble immune mediators in the onset, progression and prognosis of the main cardiovascular diseases. Long living individuals are a precious source of knowledge from which to draw not merely the secrets of healthy ageing but mainly all the mechanisms through which a correct homeostasis between immune and cardiovascular system can be reached. In the past years, we discovered that longevity-associated variant of the BPIFB4, a protein belonging to the innate immune compartment due to its antimicrobial properties, also corrects endothelial dysfunction, atherosclerosis and immunesenescence by establishing in part a protective immune response. Looking for both prognostic biomarkers as well as for innovative immunomodulatory tools, here we deeply reviewed the role of LAV-BPIFB4 in positively influencing the activity of immune cells in atherosclerosis, diabetic cardiomyopathy, and cardiovascular complications to prolonged or aberrant infections (as summarized in Table 1).

The ability of LAV-BPIFB4 to limit CVD progression and fate by a) skewing M2 response, b) reducing CD8+T cell reactivity, c) enhancing regulatory circuits in plasma (IL-10, IL-1RA), d) reducing CD38+NADase expression in immune cells, e) establishing a dual response to LPS stimulation (both enhancing and dampening) and f) enrichment of the pool of protective patrolling monocytes might suggest the best way to tune the immune system in age-related conditions (Fig. 1). Indeed, as immune cell functions that are deleterious in heart and vessels may be required for mounting an efficient host response against any kinds of insults and stressors; in future immunomodulatory signals, such as LAV-BPIFB4, more than non-specific immunosuppressants may constitute a desirable valid option in the management of CVDs.

## Figures and Tables

**Table 1:** The pleiotropic immunomodulatory activity of LAV-BPIFB4 in CVDs.

Pathology	Study Model	Type of study	LAV-BPIFB4 effect	Potential applications	Ref
COVID-19	Human PBMCs	In vitro, human recombinant LAV-BPIFB4	*Induction of biphasic immune response to SARS-CoV-2*: starting hyperactivation of CD4+ and CD8+ lymphocytes followed by its reduction in the ending phase of the treatment *Modulation of PBMCs microenvironment:* reduction of pro-inflammatory MCP-1 levels and increase of anti-inflammatory IL-1β and IL-18	Valid biomarker in COVID-19 prognosis	[49]
Diabetic cardiomyopathy	PBMCs from type 2 diabetic patients; db/db mice model	In vitro, human recombinant LAV-BPIFB4 In vivo, AAV-LAV-BPIFB4 gene therapy	*Induction of SDF-1 expression*, through the CXCR4 signaling	Promising immuno therapeutical approach in treating diabetic cardiomyopathy	[15]
Infections	LPS-infected human CD14+ monocytes; LPS-infected induced-dendritic cells	In vitro, human recombinant LAV-BPIFB4 In vitro, human recombinant LAV-BPIFB4	*Inflammatory balance recovery:* reduction of pro-inflammatory TNF-α and IL-1β levels Induction of anti-inflammatory IL-10 and TGF-β factors and expression of CD11b tolerogenic marker	Regulatory factor of myeloid cells-related immune response	[19]
Atherosclerosis	ApoE knockout mice (ApoE −/−) BMCs from atherosclerotic patients Organotypic cultures of human atherosclerotic vessels	In vivo, AAV-LAV-BPIFB4 gene therapy In vitro, human recombinant LAV-BPIFB4 Ex vivo and in vitro, human recombinant LAV-BPIFB4	*Reduction of the atherogenic process and plaque stabilization* *Peripheral monocyte redistribution and polarization* Increase of ‘classical’ Ly6C^high^ and decrease of ‘non-classical’ Ly6C^low^ circulating monocytes and shifting effect on splenic macrophages towards the pro-resolving M2 phenotype. Induction of increased peripheral blood levels of IL-23 and IL-27, key suppressor cytokines in atherosclerosis Reduction of the abundance of proliferating cytotoxic CD8+ T cells Macrophage polarization towards the alternative M2 anti-inflammatory state Less levels of pro-inflammatory IL-1β and TNF-α and increased atheroprotective IL-33 levels	Viable therapy in counteracting both vascular and immune sides of atherosclerosis	[28]
Immunesenescence	26 months C57BL/6J mice Plasma from long-living individuals and aged donors	In vivo, AAV-LAV-BPIFB4 gene therapy	Reduction of senescence in: *CD11b* + *myeloid cells* *Aortas* Increase of NAD + levels and reduction of CD38 activity Improvement of splenic catabolic activity: *increase of active NK1.1* + *CD69*+*; increase of M2 pro-resolving macrophages; reduction of spleen-resident CD38*+ *macrophages and CD38*+ *pro-inflammatory monocytes* Modulation of SASP factors in *plasma and spleen milieu* Increase of NAD + levels in LLIs	Valid senotherapeutic tool in delaying immune system senescence	[76]
